# Anxiety, Low Self-Esteem and a Low Happiness Index Are Associated with Poor School Performance in Chilean Adolescents: A Cross-Sectional Analysis

**DOI:** 10.3390/ijerph182111685

**Published:** 2021-11-07

**Authors:** Rafael Zapata-Lamana, Cristian Sanhueza-Campos, Marcia Stuardo-Álvarez, Jessica Ibarra-Mora, Marcela Mardones-Contreras, Daniel Reyes-Molina, Jaime Vásquez-Gómez, Nicole Lasserre-Laso, Felipe Poblete-Valderrama, Fanny Petermann-Rocha, Maria Antonia Parra-Rizo, Igor Cigarroa

**Affiliations:** 1 Escuela de Educación, Universidad de Concepción, Los Ángeles 4440000, Chile; rafaelzapata@udec.cl (R.Z.-L.); crsanhuezac@udec.cl (C.S.-C.); 2 Centro de Vida Saludable, Universidad de Concepción, Concepción 4030000, Chile or stuardo.marcia@gmail.com (M.S.-Á.); marcemardonesco@gmail.com (M.M.-C.); 3 Departamento de Educación Física, Deporte y Recreación, Universidad Metropolitana de Ciencias de la Educación, Santiago 7760197, Chile; jessica.ibarra@umce.cl; 4 Doctorado en Psicología, Facultad de Ciencias Sociales, Universidad de Concepción, Concepción 4030000, Chile; danielreyes@udec.cl; 5 Centro de Investigación de Estudios Avanzados del Maule (CIEAM), Universidad Católica del Maule, Talca 3460000, Chile; jvasquez@ucm.cl; 6 Laboratorio de Rendimiento Humano, Grupo de Estudios en Educación, Actividad Física y Salud (GEEAFyS), Universidad Católica del Maule, Talca 3460000, Chile; 7 Escuela de Nutrición y Dietética, Facultad de Salud, Universidad Santo Tomás, Los Ángeles 4440000, Chile; nlasserre@santotomas.cl; 8 Facultad de Educación, Departamento de Ciencias del Deporte y Acondicionamiento Físico, Universidad Católica de la Santísima Concepción, Concepción 4030000, Chile; fpoblete@ucsc.cl; 9 Facultad de Medicina, Universidad Diego Portales, Santiago 8370068, Chile; fanny.petermann@mail.udp.cl or; 10 Institute of Cardiovascular and Medical Sciences & Institute of Health and Wellbeing, University of Glasgow, Glasgow G12 8TA, UK; 11 Department of Health Psychology, Faculty of Social and Health Sciences, Campus of Elche, Miguel Hernandez University (UMH), 03202 Elche, Spain; maria.parrar@umh.es; 12 Escuela de Kinesiología, Facultad de Salud, Universidad Santo Tomás, Los Ángeles 4440000, Chile

**Keywords:** mental health, anxiety, self-esteem, happiness, subjective well-being, school performance, teenage students

## Abstract

Objective: To analyze the relationship between anxiety, self-esteem, happiness index and primary school students’ academic performance in Chilean adolescents from the Biobío province. Methodology: 733 (46.1% girls; 12 (1.3 years)) public primary school students that completed the 2018 Health and School Performance Survey carried out in the Biobío province were included in this cross-sectional analysis. The BECK Anxiety Inventory (BAI) was used to measure anxiety while happiness index and self-esteem were measured using the subjective happiness scale and the Rosenberg self-esteem scale, respectively. School performance was measured by grade point average (GPA) of language, math, physical education and cumulative GPA, and behavior associated with cognition in the school context was also considered. The relationship between mental health indicators and school performance was investigated using a one-way ANOVA and Pearson correlation. Results: In comparison to students with low anxiety levels and high self-esteem and happiness levels, students with higher anxiety levels, lower self-esteem and happiness levels perceived themselves as having memory problems. They were also slower to solve math problems, had a shorter attention span in class and presented more difficulties in solving complex tasks, as well as being more nervous during testing. These students also got the lowest grade point average in math, language and physical education. Conclusions: High anxiety levels, low self-esteem and low happiness levels were associated with lower school performance and weaker behavior associated with cognition in Chilean adolescents. Implementing plans of emotional education and mental health could improve academic achievement.

## 1. Introduction

High-quality education is the ethical threshold by which those who work in teaching operate daily. Nowadays, new skills and knowledge demands are imposed on the educational system to prepare students to face the challenges of a complex society [1]. Yet, having a broad curriculum does not necessarily translate into more quality and integral education. In fact, an inclusive educational system should contain all developmental areas of a person, among them affective, artistic, spiritual, physical and moral dimensions [2].

In recent years, the students’ socio-affective competencies and emotional intelligence—particularly their impact on school performance—have been two fields of interest for educational specialists [3]. Previous studies in these areas have shown that student anxiety, self-esteem and depression are related to the concept of well-being and—more broadly—to the mental health concept accepted by the World Health Organization (WHO) [4]. In this regard, anxiety is an aversive psychological state that occurs when the level of perceived threat is considered high [5]. The latter can have physiological [6], cognitive [7], behavioral and emotional effects on people [8,9], affecting academic performance, especially during demanding tasks in terms of attention [5,10]. Self-esteem is a psychological construct defined as a person’s positive or negative attitude towards himself from a positive or negative personal experience [11]. Happiness, in turn, is the general evaluation that an individual has about life based on self-determined standards [12,13]. Better happiness is essential to the overall development of an individual, and when life in general is good, people will feel happy and well [12,13].

However, the well-being concept is wide. In terms of educational area, this is related to the interaction of different aspects, including motivation, attitudes, decision-making, and learning tasks [14]. Recently, special attention has been placed on studies linking well-being with school performance at university and school levels [15,16,17]. The latter reveals a relationship between high well-being levels and the setting of reachable goals as well as between learning and grade point average [15].

At the moment, grade point averages (GPAs) are the most stable predictor to assess the relationship between well-being and school performance [3]; the adequate measurement of which is done through different strategies [4]. In addition to this, children use cognitive abilities related to everyday tasks such as memory, speed and attention that heavily influence school performance and social adaptation; these abilities are connected to the operational attention and emotional regulation system [18].

Every day, students are exposed to several school activities for which they are demanded to develop answers, decide, create and execute the process of attention, planning, memorizing, language, and flexibility [18,19,20]. Special attention must be paid to math [15], physical education [21] and language, which have proved to have a strong relationship with well-being indicators and school performance [22,23].

Evidence reinforces the relevance of the self-perception of happiness [24], not only because happier students learn better, but because they have more confidence and feel more accepted and valued [25]. The latter leads to stronger self-esteem. Additionally, those with higher self-concept perceptions have greater satisfaction and affection in life than those with a lower perception [24].

Given the above, more research on classroom strategies that would impact school performance and promote favorable practices is more necessary than ever. These favorable practices could improve students’ general well-being and reduce the stress level in the classroom, which, in turn, would facilitate the integration into richer school environments and allow for the development of socioemotional competencies most needed in today’s society [25]. Jonberg, Pereira and Kastens [26] point out the relationships between anxiety and school performance. He demonstrated that children with lower academic self-concepts showed higher anxiety levels, with anxiety negatively predicting future achievements [26]. Likewise, other authors have also suggested the need to study the negative effects of anxiety on school performance [27] since students with higher anxiety levels are likely to learn less [28]. However, the association between happiness and academic performance is an emerging area of research, and, unfortunately, there is limited evidence on this relationship in children and adolescents [29,30].

A recent study carried out in Chile showed how low academic performance is associated with these types of problems [31]. One example is school dropout [31], which can have significant public health costs [32,33] due to its relationship with illicit substances [34], psychological health issues [35], or teenage pregnancy [36]. In Chile, education is mandatory from five to eighteen years old. Overall, 14 grades: eight during primary education and four during secondary education [37]. Primary education is aimed at children from the age of six, and consists of ten subjects, with a total study load of 38 hours per week [37].

Despite the above, current research efforts mainly focus on the university population, with Europe and the United States leading this research field [25]. However, research in Latin American countries on this population is lacking. Even if few, a previous longitudinal study carried out in Chilean schoolchildren showed that mental health was a significant predictor of academic performance [38]. Nevertheless, no line of research currently interconnects the variables of anxiety, self-esteem and happiness index, nor has it studied whether they develop independently or are associated with psychological aspects of other students [25,39].

Therefore, this study aimed to analyze the relationship between anxiety, self-esteem, happiness index and school students’ academic performance in Chilean adolescents from the Biobío province.

## 2. Materials and Methods

### 2.1. Design and Participants

This cross-sectional study used data from the Health and School Performance Survey carried out in the Biobío province in 2018. The survey included all 5th to 8th-grade students from the four public schools in one city of the Biobío providence (*n* = 3857). From this universe, a probabilistic and stratified sample of 797 (12 ± 1.3 years) were included. After parents/legal guardians consented to be part of the study, the participants in the sample completed all the measurements. Sixty-four students were excluded for not meeting the inclusion criteria (survey or consent), so the final sample was 733 students (46.1% girls). The sample calculation considered a 5% margin of error and a 95% confidence level.

### 2.2. Procedure and Instruments

An agreement between the university research team and the municipal management and education department was reached to facilitate collaborative work between the parties. The study’s design and variables selection were carried out in conjunction with the schools’ teachers and administrators. Then, a group of teachers was trained to administer the tests to minimize the inter-rater risk of bias. The data elicitation procedure took place on the same day and time. All instruments were applied under confidentiality protocols and duly endorsed by the Ethics Committee of Universidad de Concepción, Chile, the corresponding municipality and following the Declarations of Helsinki and Singapore.

*Anxiety level*: The BECK Anxiety Inventory (BAI), Spanish version was used [40]. This instrument consists of 21 questions for teenagers and adults, which describe emotional, physiological and cognitive symptoms of anxiety. Each question has four possible answers to measure the severity of the current anxiety. Scoring goes from 0 to 63, ranging from very low anxiety (0–21 points); moderate (22–35 points); and severe anxiety (36 points and above). The estimated time for test completion is about 5 to 10 min. One of the items asks about mood, pessimism, feelings of failure. High indices of internal consistency have been found in the Spanish validation of the questionnaire of α = 0.83 and reliability of ω = 0.72 [41], and in the Chilean population an internal consistency of α = 0.90 [42]

*Self-esteem*: The Rosenberg self-esteem scale (SES), Spanish version was used to measure it [43]. This scale determines the person’s feelings of respect and acceptance. The Rosenberg scale has been widely used in children and teenager studies [44,45,46] and consists of 10 items with a 4-point Likert-type scale, proportionately divided into positive and negative statements. Participants must answer according to the level of agreement with the statement. Scoring goes from 10 to 40, ranging from low self-esteem (25 points), moderate (26–29 points), to high self-esteem (30 points and above). The estimated time for test completion is about 5 to 10 min [43]. Some examples of items are “in general I am inclined to think that I am a failure”; “I am capable of doing things as well as most people”; “I feel like I don’t have much reason to be proud”; or “sometimes I feel really useless”. The SES has been previously validated in Chile, presenting an internal consistency of α = 0.74 and a reliability of ω = 0.75 [47], being used in the Chilean school population presenting a consistency of α = 0.84 [48].

*Perception of happiness*: The Subjective Happiness Scale (SHS) was used to measure it [49]. This scale includes four items with a 7-point Likert-type scale. Following reverse coding of the fourth item, a single composite score is computed by averaging the responses to the four items. Scoring ranges from 1.0 to 7.0, with higher scores reflecting greater happiness. The estimated time for test completion is about 5 min. Some examples of items are “in general I consider myself”; “compared to most of my peers, I consider myself”; (not a very happy person or a very happy person). The SHS has shown an internal consistency greater than α = 0.73 and a reliability of ω = 0.73 [50], being previously used in Chile, presenting a consistency of α = 0.83 [51].

*School Performance*

*Behavior associated with cognition in the school context:* A survey was designed by the study’s researchers to measure this dimension, including five items from the Scale of Daily Stress was applied [52]. The questions were: How good is your memory? How fast can you solve a math problem? How long can you pay attention in class? How well can you solve complex tasks in the school? How nervous do you feel during a test? Scoring went from 0 to 10, with lower scores reflecting problems with the behavior being measured. Additionally, the responses were categorized into low (0–3), medium (4–6) and high (7–10). This questionnaire has already been used in recent studies to evaluate behaviors associated with cognition in the school context [53].

*Grade point average (GPA)*: This included the grades on language, math, physical education subjects and the cumulative GPA of an academic semester. The Chilean grading system goes from one to seven, where four is the minimum passing grade. All the participating schools followed the same Ministry of Education curriculum and study plans, so there were no differences in the academic demands [54].

*Socio-educational data*: Additionally, age, sex, school grades and special needs education participation were also elicited to better characterize the sample.

### 2.3. Statistical Analyses

A descriptive analysis was carried out, including both the socio-educational and mental health variables. The qualitative data were represented by frequency and percentage, while the quantitative data by the mean and standard deviation. Data distribution was established by means of normality and equality variance tests (Shapiro-Wilk and Levene). To establish the association between nominal variables, the Chi-square test was used. The difference in means between two different groups was tested with the independent samples T-Student test. To determine the effect of anxiety, self-esteem and happiness index on GPA and behavior associated with cognition in the school context were determined by one-way ANOVA. To establish significant differences between the categories of anxiety, self-esteem and happiness index, the Bonferroni Post hoc test was performed. Finally, to establish the linear relationship between anxiety, self-esteem and happiness index and the variables of school performance/cognition associated behaviors in the school context, Pearson’s correlation coefficient was used. Cohen’s d effect size (ES) was calculated and qualitatively assessed as trivial (0–0.19), small (0.20–0.49), medium (0.50–0.79), or large (0.80 and greater). The association of anxiety, self-esteem and happiness index and behavior associated with cognition in the school context and grade point average were investigated using linear regression analyses. Data are presented as β-coefficient and its 95% CI. Multivariable models were adjusted for relevant confounders. Model 0—unadjusted; and Model 1—adjusted socio-educational variables (sex, school grade and special needs integration program). All analyses were performed using SPSS version 25.0 software (IBM statistics, Chicago, IL, USA). Significance at the level of *p* < 0.05 was used.

## 3. Results

### 3.1. General Characteristics of the Study Population

Table 1 shows the general characteristics of the study population, including the behavior associated with cognition and the participants’ grade point average. Overall, boys represented 53.9% of the sample with an average age of 12 years; 19.2% of the students belonged to the special needs education program (22.3% boys vs. 15.7% girls (*p* = 0.024). In the descriptive analysis, the schoolchildren mostly reported to have good memory use in class (60.4%); be fast at solving math problems (50,6%); have a moderate attention span (40.9%); have no problems solving complex tasks (47.1%); and feel at ease during testing (52%). It is noted that 22% of schoolchildren are perceived as very nervous during tests, 19.5% have poor attention span in class and 16.5% are slow to solve math problems. When the association of behaviors associated with cognition in the school context was measured according to sex through the chi-square test, it was observed that a small proportion of boys perceived themselves slower at solving math problems, found complex tasks very difficult and felt very nervous in tests compared to girls (*p* = 0.024, *p* = 0.040 and *p* = 0.015, respectively). On the other hand, when the differences in means between boys and girls were compared through the student’s T-test, it was evidenced that girls showed better cumulative GPA and language GPA than boys (*p* = 0.002 and *p* = 0.007, respectively).

### 3.2. Anxiety, Self-Esteem and Happiness Index

Table 2 shows the participants’ mental health indicators by sex. The difference in means between boys and girls in anxiety, self-esteem and happiness index was evaluated with the T-student test. No significant differences were found by sex. Additionally, when sex and anxiety, self-esteem and happiness index classifications were associated through chi-square, no significant association was found. However, 58.8% of the participants reported having medium or low self-esteem, while 67% expressed having a medium or low perception of happiness, and 15.6% presented moderate to severe anxiety levels.

### 3.3. Behavior Associated with Cognition in the School Context According to Anxiety, Self-Esteem and Happiness Index

When the ANOVA analysis was performed, significant differences were found between the three groups in five types of behavior associated with cognition in the school context. Thus, in anxiety level (Memory use in class (*p* < 0.0001; with a medium effect size); Speed to solve math problems (*p* < 0.0001; with a small effect size); Attention span (*p* = 0.003; with a trivial effect size); Solution of complex tasks (*p* < 0.0001; with a small effect size); Nervousness during testing (*p* = 0.003; with a trivial effect size).Then, in self-esteem level (Memory use in class (*p* < 0.0001; with a medium effect size); Speed to solve math problems (*p* < 0.0001; with a medium effect size); Attention span (*p* < 0.0001; with a large effect size); Solution of complex tasks (*p* < 0.0001); Nervousness during testing (*p* = 0.009; with a trivial effect size) and in happiness index, (Memory use in class (*p* < 0.0001; with a medium effect size); Speed to solve math problems (*p* < 0.0001; a small effect size); Attention span (*p* < 0.0001; with a medium effect size); Solution of complex tasks (*p* < 0.0001; with a medium effect size); Nervousness during testing (*p* = 0.011; with an effect size trivial). Upon closer analysis, when comparing the groups with the Bonferroni test, those schoolchildren categorized as showing severe anxiety perceived themselves as having poorer memory skills than the other groups (*p* < 0.0001). The group with moderate to severe anxiety perceived themselves as slower to solve math problems (*p* < 0.0001), with greater difficulty paying attention in class (*p* < 0.0001), solving complex tasks (*p* < 0.0001) and felt more nervous when taking tests (*p* < 0.0001). Concerning the perception of happiness, the participants categorized as having a low perception of happiness perceived themselves as slower at solving math problems (*p* < 0.0001), with greater difficulty paying attention in class (*p* < 0.0001), solving complex tasks (*p* < 0.0001) and felt more nervous (*p* < 0.0001) when taking tests compared to the other groups. Furthermore, those participants with moderate to low perceptions of happiness perceived themselves as having poorer memory skills in class (*p* < 0.0001) than those with a high perception (Table 3). Regarding the participants’ self-esteem, those categorized with a low perception of self-esteem perceived themselves as having bad memory in class (*p* < 0.0001), with more difficulties when solving complex tasks (*p* < 0.0001), slower in solving math problems (*p* < 0.0001), and more nervous during tests (*p* < 0.0001) than the other groups (Table 3).

### 3.4. Students’ Grade Point Average (GPA) According to Anxiety, Self-Esteem and Happiness Index

According to the participants‘ anxiety level, significant differences were found between the three groups in the GPA through ANOVA analysis. Thus, in anxiety level (GPA of math (*p* = 0.017; with a trivial effect size); GPA in physical education ((*p* < 0.0001; with a small effect size) and the cumulative GPA (*p* = 0.026; with a trivial effect size). Then, in self-esteem (GPA of math (*p* < 0.0001; with an effect size small); GPA in Language (*p* < 0.0001; with a small effect size); GPA in physical education (*p* = 0.002; with a trivial effect size) and the cumulative GPA (*p* < 0.0001; with a medium effect size) and in happiness index (GPA in physical education (*p* = 0.003; with a trivial effect size) and the cumulative GPA (*p* = 0.016; with a trivial effect size). Upon closer analysis, when comparing the groups with the Bonferroni test, the participants with moderate and severe anxiety got a lower GPA in physical education than those having very low anxiety (*p* = 0.008). Then, the participants with a low perception of happiness got a lower GPA in physical education and cumulative GPA compared to those with a high and moderate perception of happiness (*p* = 0.001). Finally, schoolchildren with a lower perception of self-esteem got a lower GPA in math (*p* < 0.0001) and physical education (*p* < 0.0001) than those who showed a moderate to a higher perception of self-esteem. Additionally, schoolchildren with a moderate-to-low perception of self-esteem got the lowest grade point average in language (*p* < 0.0001) and in the cumulative GPA (*p* < 0.0001) (Table 4).

### 3.5. Correlations between Anxiety, Self-Esteem and Happiness Index and School Performance

The linear association between anxiety, self-esteem and happiness index and behavior associated with cognition in the school context and GPA was analyzed with Pearson’s linear correlation coefficient and Cohen’s d effect size (ES) was calculated. Significant correlations were found in all parameters. Participants with higher levels of anxiety, lower perception of happiness and lower self-esteem perceived to have more memory problems in class, more difficulties in solving math problems, more difficulties in paying attention, felt more nervous during testing and complex tasks were harder to solve, although the effect size was trivial or small. As for the GPA, those participants with higher levels of anxiety and lower self-esteem got—on average—lower cumulative GPA and lower GPA in math, language, and physical education. Similar results were found for lower levels of happiness but math. In these cases, the effect size was also trivial or small (Table 5).

### 3.6. Association between Anxiety, Self-Esteem and Happiness Index and Behavior Associated with Cognition in the School Context and GPA

The association between anxiety, self-esteem and happiness index and behavior associated with cognition in the school context and GPA was estimated by linear regression analysis. For the unadjusted model, schoolchildren with very low anxiety were associated with better memory use in class, higher attention span and solution of complex tasks than schoolchildren with severe anxiety. In the same line, it was observed that schoolchildren with low and moderate perceptions of self-esteem and happiness were associated with lower memory use in class, slower to solve math problems, lower attention span, solution of complex tasks, more difficulty for resolution of complex tasks and more nervous during testing compare to schoolchildren with a high perception of self-esteem and happiness. After adjusting the model, similar associations were maintained for socio-educational variables (sex, school grade and special needs integration program) (Table 6).

In addition, in the unadjusted linear regression model, schoolchildren with very low anxiety were associated with better math and physical education GPA than schoolchildren with severe anxiety. Schoolchildren with low and moderate perceptions of self-esteem were associated with lower math, language, and cumulative GPA. Schoolchildren with a low perception of self-esteem were associated with lower physical education GPA compared to schoolchildren with a high perception of self-esteem. Additionally, schoolchildren with lower and moderate perceptions of happiness were associated with a lower cumulative GPA. Schoolchildren with a lower perception of happiness were associated with a lower physical education GPA compared with schoolchildren with a high perception of happiness. After adjusting the model, similar associations were maintained for socio-educational variables (sex, school grade and special needs integration program) (Table 7).

## 4. Discussion

The main findings of this study suggest that 58.8% and 57% of the participants had low-to-moderate self-esteem and perception of happiness, respectively, while 15.6% reported having moderate-to-high anxiety levels. Participants with higher anxiety levels and lowers happiness and self-esteem levels perceived themselves as having poorer memory skills, being slower at solving math problems, having more difficulties solving complex tasks and paying attention in class. They were also more nervous during a test than participants with lower anxiety levels and higher happiness and self-esteem levels.

In general, participants with higher anxiety levels and lower happiness and self-esteem levels got—on average—lower cumulative GPA and lower GPA in math, language and physical education than those with lower anxiety levels and higher happiness and self-esteem perceptions of themselves. Concerning anxiety, results showed that 84.8% of the participants presented very low anxiety levels. Previous studies have demonstrated that the higher the anxiety levels, the lower the school performance [55,56,57,58]. Therefore, considering the use of adaptative strategies of emotional regulation could lower anxiety levels and favor school performance [59]. Our findings are also in line with the results by Hyseni Duraku and Hoxha [60], who examined the relationship between anxiety, self-esteem and school performance in secondary and university students, identifying a relationship between lower anxiety levels and higher academic performance. This is also supported by Aritzeta, Soroa [61], who found similar results by means of a program to reduce stress in university students.

This study also found that 4 out of 10 participants reported high levels of self-esteem. In this respect, previous evidence suggests that adolescents that have a heightened perception of self-esteem have better stress management, better response to failure, higher persistence in complex tasks and, in general, better response to schoolwork [62,63,64]. In contrast, having lower self-esteem has been associated with poorer school performance [65].

It has been observed that happiness’s moderating effect is a positive element for academic achievement; the greater the happiness, the higher the prediction degree of academic achievement [66]. The present study found that 3 out of 10 students had a high perception of happiness, which eventually proved to be related to higher levels of self-efficacy, satisfaction and psychological well-being [67]. In contrast, lower levels of well-being perceived by some participants may be due to personal, contextual and physical factors [68] present in the form of defensive and evasive strategies [69]; low socio-economic status [70]; lack of coping strategies or abilities to face schoolwork successfully [4]; or lack of social support [71].

Regarding behavior associated with cognition, 39.5% of the participants judged their memory to be moderate or bad. Cognitive abilities explain teenagers’ different learning and academic performance levels in the school stage [72]. Memory is related to high academic performance. In specific areas [73,74], it is considered essential for the development of higher cognitive functions [75] and can be enhanced through training and practice [76]. Memory and attention are the main neuro-psychological capacities that support the learning processes [77]. Attention allows students to select, process and solve sensory stimuli and, recruit and activate brain areas to provide the appropriate response [78]. In the present study, 39.6% of the participants considered their attention span good, whereas 60.4% considered it bad or moderate. This is particularly relevant as attention can be a risk factor for the disorder of certain cognitive abilities such as processing speed, temporal processing and working memory in reading and math [79]. Due to its importance in the educational process, different strategies have been successfully developed, mainly based on physical activity, to improve selective attention and reduce inattention time [80,81]. As stated above, attention is related to processing speed, which is considered by some researchers a predictor of reasoning development [82] and academic achievement [83,84,85]; it can also be used to detect attention deficit disorder [86]. The participants of the present study who perceived their speed to solve math problems as low or moderate correspond to 49.4%; this perception could have probably been influenced by mathematics anxiety as an underlying factor [87], which could be related to the 48% of the participants who reported feeling nervous during testing. Similarly, solving complex tasks is considered a metacognitive strategy, or a skill of high cognitive order, which involves several variables [88,89] and whose training from early ages is effective in the acquisition of self-regulation and self-evaluation metacognitive strategies [90]. Of the present study participants, 52.9% perceive this ability as difficult in different levels, being probably affected by various executive function components, even beyond the calculating knowledge and reading skills [91].

### 4.1. Strengths and Limitations

This study used a representative sample and is the most recent research in Chile giving an account of the relationship between mental health indicators such as anxiety, self-esteem and happiness and the academic performance of primary school students. There are no previous studies in Chilean adolescents that report an association between the variables described and the questionnaires used were previously validated. However, it is not exempt from limitations. Firstly, low school performance is multifaceted, and, therefore, this study’s results cannot be attributed exclusively to the participants’ mental health and/or well-being. In this sense, the present study did not analyze other intervening variables in school performance such as personal, family or contextual factors. Future studies could more closely examine the behavior or current lifestyles that may play a role in school performance, such as cell phones, low levels of physical activity, dietary habits, or sleeping routines. Finally, due to the cross-sectional nature of this study, causality cannot be inferred.

### 4.2. How Does This Literature Review Contribute to the Existing One?

The present study results show that participants with higher anxiety levels and lower happiness and self-esteem levels perceived themselves as having a bad memory, being slower at math problem solving, having a shorter attention span, having more difficulties solving complex tasks, and feeling more nervous during testing. In addition, it was also noted that participants with higher anxiety levels and lower happiness and self-esteem levels got—on average—lower grade point averages and lower average grades in math, language and physical education.

## 5. Conclusions

These findings contribute to the local information and support existing evidence relating to lower levels of self-esteem, happiness index, higher anxiety levels, and lower school performance. Thus, it becomes the most up-to-date evidence that accounts for the relationship between anxiety, self-esteem, happiness, and schoolchildren’s school performance from Chilean public educational centers. Additionally, this study is a pioneer in Latin America in associating the perception of happiness and school performance, opening a promising research line on happiness and academic performance, which will complement the current evidence provided from the perspective of positive psychology on the relationship of these variables [12,13].

Moreover, this research paper sheds light on the importance of mental health studies, particularly from the perception of happiness and their implications on school performance and the behavior associated with cognition in school settings. These results can provide evidence and be used in educational centers to reflect on the importance of mental health in schoolchildren and on the potential negative impact of lower levels of self-esteem, happiness and higher anxiety levels on school performance and related behaviors with cognition in the school environment. Additionally, this study provides evidence to continue with the integration of interventions that promote better psychological health and academic performance in schoolchildren [7,29,30,38].

Therefore, this study brings about the need to promote programs that directly impact mental health indicators and allow school students to improve their academic performance. Finally, these findings also reinforce the need to develop emotional education plans and promote general mental health in school students, as they could be directly related to school performance [35,36]. Based on these conclusions, it is possible to assert that a formal educational process must balance theory and practice to develop a socially constructed knowledge system that includes teenagers and their perceptions [91].

## Figures and Tables

**Table 1:** Socio-educational characteristics, behavior associated with cognition in the school context and grade point average by sex.

Variables	Boys	Girls	All Schoolchildren
*n (%)*	395 (53.9)	338 (46.1)	733 (100)
*Age (years),mean (SD)*	12.1 (1.4)	12.0 (1.2)	12.0 (1.3)
*School grade*			
*Fifth*	94 (23.8)	68 (20.1)	162 (22.1)
*Sixth*	98 (24.8)	89 (26.3)	187 (25.5)
*Seventh*	96 (24.3)	102 (30.2)	198 (27.0)
*Eighth*	107 (27.1)	79 (23.4)	186 (25.4)
*Special needs integration program*			
*Yes*	88 (22.3)	53 (15.7) ^⸸^	141 (19.2)
*No*	307 (77.7)	285 (84.3)	592 (80.8)
Behavior associated with cognition in the school context			
*Memory use in class*			
*Bad*	18 (4.6)	30 (8.9)	48 (6.5)
*Moderate*	133 (33.7)	109 (32.2)	242 (33.0)
*Good*	244 (61.8)	199 (58.9)	443 (60.4)
*Speed to solve math problems*			
*Slow*	44 (11.1)	77 (22.8) ^⸸^	121 (16.5)
*Moderate*	124 (31.4)	117 (34.6)	241 (32.9)
*Fast*	227 (57.5)	144 (42.6)	371 (50.6)
*Attention span*			
*Bad*	71 (18.0)	72 (21.3)	143 (19.5)
*Moderate*	162 (41.0)	138 (40.8)	300 (40.9)
*Good*	162 (41.0)	128 (37.9)	290 (39.6)
*Solution of complex tasks*			
*‘It’s very hard’*	45 (11.4)	64 (18.9) ^⸸^	109 (14.9)
*‘It’s somewhat hard’*	146 (37.0)	133 (39.3)	279 (38.1)
*‘It’s not hard’*	204 (51.6)	141 (41.7)	345 (47.1)
*Nervousness during testing*			
*Very nervous*	72 (18.2)	89 (26.3) ^⸸^	161 (22.0)
*Moderately nervous*	101 (25.6)	90 (26.6)	191 (26.1)
*Not nervous*	222 (56.2)	159 (47.0)	381 (52.0)
Grade point average (GPA)			
*Math (1.0–7.0), M (SD)*	5.2 (1.00)	5.2 (1.03)	5.2 (1.01)
*Language (1.0–7.0), M (SD)*	5.0 (0.82)	5.2 (0.80) *	5.1 (0.82)
*Physical education (1.0–7.0), M (SD)*	6.4 (0.55)	6.4 (0.53)	6.4 (0.54)
*Cumulative GPA (1.0–7.0), M (SD)*	5.6 (0.57)	5.7 (0.59) *	5.6 (0.58)

Qualitative data are presented by frequency and percentages; quantitative data is presented by mean (standar desviation) M (SD). Means difference between boys and girls was tested with the independent samples T-Student test. * *p* < 0.05. To examine the association between sex and anxiety, self-esteem and happiness index the Chi-square test was used. ^⸸^ *p* < 0.05. *n* = 733.

**Table 2:** Anxiety, self-esteem and happiness index in school students by sex.

Variables	Boys	Girls	All Schoolchildren
Anxiety			
*Beck anxiety inventory (0–63 points)*	13.4 (8.3)	13.9 (7.8)	13.6 (8.1)
*Low anxiety*	343 (86.9%)	276 (81.7%)	619 (84.4%)
*Moderate anxiety*	44 (11.1%)	57 (16.9%)	101 (13.8%)
*Severe anxiety*	8 (2.0%)	5 (1.4%)	13 (1.8%)
Self-esteem			
*Rosenberg self-esteem scale (10–40 score)*	28.8 (4.7)	28.8 (4.7)	28.8 (4.7)
*High perception of self-esteem*	164 (41.5%)	138 (40.8%)	302 (41.2%)
*Moderate perception of self-esteem*	124 (33.9%)	116 (34.3%)	250 (34.1%)
*Low perception of self-esteem*	97 (24.6%)	84 (24.9%)	181 (24.7%)
Happiness			
*Subjective happiness scale (1–7 points)*	5.2 (1.0)	5.2 (1.1)	5.2 (1.1)
*High perception of happiness*	128 (32.4%)	114 (33.7%)	242 (33.0%)
*Moderate perception of happiness*	193 (48.9%)	152 (45.0%)	345 (47.1%)
*Low perception of happiness*	74 (18.7%)	72 (21.3%)	146 (19.9%)

Qualitative data are presented by frequency and percentages; quantitative data is presented by mean (standard deviation) M (SD). Means difference between boys and girls was tested with the independent samples T-Student test. To examine the association between sex and anxiety, self-esteem and happiness index the Chi-square test was used. *n* = 733.

**Table 3:** Behavior associated with cognition in the school context by anxiety, self-esteem and happiness index.

	Anxiety					
Behavior Associated with Cognition in the School Context	Very Low	Moderate	Severe	F	*p*-Value	ES
*Memory use in class*	7.1 (2.0) ^a^	6.1 (2.6) ^b^	4.3 (2.8) ^c^	19.998	<0.0001 **	0.52
*Speed to solve math problems*	6.5 (2.5) ^a^	5.3 (2.9) ^b^	5.6 (2.8) ^a^	10.296	<0.0001 **	0.27
*Attention span*	5.8 (2.4) ^a^	5.0 (2.7) ^b^	4.3 (2.6) ^b^	5.883	0.003 **	0.16
*Solution of complex tasks*	6.3 (2.3) ^a^	5.4 (3.7) ^b^	4.7 (3.4) ^b^	8.600	<0.0001 **	0.23
*Nervousness during testing*	6.4 (2.8) ^a^	5.4 (3.4) ^b^	5.5 (3.5) ^a^	5.846	0.003 **	0.16
	Self-esteem					
	High perception	Moderate perception	Low perception	F	*p*-value	ES
*Memory use in class*	7.5 (1.9) ^a^	6.9 (2.1) ^b^	6.1 (2.3) ^c^	27.871	<0.0001 **	0.71
*Speed to solve math problems*	7.0 (2.4) ^a^	6.1 (2.5) ^b^	5.3 (2.7) ^c^	25.999	<0.0001 **	0.66
*Attention span*	6.3 (2.4) ^a^	5.6 (2.2) ^b^	4.6 (2.5) ^c^	32.337	<0.0001 **	0.81
*Solution of complex tasks*	6.8 (2.3) ^a^	6.0 (2.3) ^b^	5.3 (2.5) ^c^	25.595	<0.0001 **	0.61
*Nervousness during testing*	6.6 (2.8) ^a^	6.2 (2.9) ^a^	5.7 (3.0) ^b^	4.793	0.009 **	0.13
	Happiness					
	High perception	Moderate perception	Low perception	F	*p*-value	ES
*Memory use in class*	7.3 (2.0) ^a^	7.1 (2.0) ^b^	5.9 (2.3) ^b^	24.209	<0.0001 **	0.62
*Speed to solve math problems*	6.8 (2.5) ^a^	6.3 (2.5) ^b^	5.3 (2.6) ^c^	16.739	<0.0001 **	0.44
*Attention span*	6.2 (2.5) ^a^	5.6 (2.3) ^b^	4.7 (2.4) ^c^	19.174	<0.0001 **	0.50
*Solution of complex tasks*	6.7 (2.2) ^a^	6.1 (2.4) ^b^	5.1 (2.5) ^c^	22.442	<0.0001 **	0.58
*Nervousness during testing*	6.5 (2.8) ^a^	6.3 (2.8) ^a^	5.6 (3.1) ^b^	4.522	0.011 *	0.12

Statistical analysis was performed with one-way ANOVA. ^abc^ Mean within a row with a different symbol indicates statistically significant difference between groups (one-way ANOVA and post hoc comparison with Bonferroni test). ** = Significance at the level *p* < 0.01. * = Significance at the level *p* < 0.05. ES: effect size.

**Table 4:** Grade point average according to anxiety, self-esteem and happiness index.

Anxiety						
Grade Point Average	Very Low	Moderate	Severe	F	*p*-Value	ES
*Math (1.0–7.0)*	5.2 (1.0)	5.0 (1.0)	4.6 (1.0)	4.113	0.017 *	0.11
*Language (1.0–7.0)*	5.1 (0.8)	5.0 (0.8)	4.9 (0.6)	0.537	0.584	0.01
*Physical education (1.0–7.0)*	6.4 (0.5) ^a^	6.2 (0.7) ^b^	6.0 (0.9) ^b^	7.792	<0.0001 **	0.21
*Cumulative GPA (1.0–7.0)*	5.7 (0.6)	5.5 (0.6)	5.4 (0.6)	3.678	0.026 *	0.10
Self-esteem						
	High Perception	Moderate perception	Low perception	F	*p*-value	ES
*Math (1.0–7.0)*	5.4 (1.0) ^a^	5.1 (1.0) ^b^	4.9 (1.0) ^c^	12.964	<0.0001 **	0.34
*Language (1.0–7.0)*	5.3 (0.8) ^a^	5.0 (0.8) ^b^	4.9 (0.8) ^b^	15.002	<0.0001 **	0.39
*Physical education (1.0–7.0)*	6.5 (0.4) ^a^	6.4 (0.5) ^a^	6.3 (0.7) ^b^	6.464	0.002 **	0.17
*Cumulative GPA (1.0–7.0)*	5.8 (0.6) ^a^	5.6 (0.6) ^b^	5.5 (0.6) ^b^	19.716	<0.0001 **	0.51
Happiness						
	High Perception	Moderate perception	Low perception	F	*p*-value	ES
*Math (1.0–7.0)*	5.2 (1.0)	5.2 (1.1)	5.0 (1.0)	1.417	0.243	0.04
*Language (1.0–7.0)*	5.2 (0.8)	5.1 (0.8)	5.1 (0.8)	2.270	0.104	0.06
*Physical education (1.0–7.0)*	6.4 (0.5) ^a^	6.4 (0.5) ^a^	6.3 (0.7) ^b^	5.724	0.003 **	0.15
*Cumulative GPA (1.0–7.0)*	5.7 (0.5) ^a^	5.6 (0.6) ^a^	5.6 (0.7) ^b^	4.129	0.016 *	0.11

Statistical analysis was performed with one-way ANOVA. ^abc^ Mean within a row with a different symbol indicates statistically significant difference between groups (one-way ANOVA and post hoc comparison with Bonferroni test). Cohen’s d effect size (ES) was calculated and qualitatively assessed as trivial (0–0.19), small (0.20–0.49), medium (0.50–0.79), or large (0.80 and greater). ** = Significance at the level *p* < 0.01. * = Significance at the level *p* < 0.05. ES: effect size.

**Table 5:** Linear correlation between anxiety, self-esteem and happiness index and behavior associated with cognition in the school context and grade point average.

	Behavior Associated with Cognition in the School Context						Grade Point Average (GPA)			
Variables		1	2	3	4	5	Math	Language	P.E.	C. GPA
*Anxiety*	*r*	−0.245 **	−0.184 **	−0.211 **	−0.207 **	−0.166 **	−0.131 **	−0.090 *	−0.121 **	−0.131 **
*Self-esteem*	*r*	0.313 **	0.301 **	0.345 **	0.293 **	0.141 **	0.211 **	0.244 **	0.128 **	0.266 **
*Happiness*	*r*	0.285 **	0.243 **	0.263 **	0.270 **	0.134 **	0.072	0.079 *	0.133 **	0.119 **

1 = Memory use in class, 2 = Speed to solve math problems, 3 = Attention span, 4 = Solution of complex tasks, 5 = Nervousness during testing. P.E. = Physical education. C. GPA = Cumulative GPA. Linear relationship between anxiety, self-esteem and happiness index and the variables of school performance/cognition associated behaviors in the school context, Pearson’s correlation coefficient was used. Cohen’s d effect size (ES) was calculated and qualitatively assessed as trivial (0–0.19), small (0.20–0.49), medium (0.50–0.79), or large (0.80 and greater). ** = Significance at the level *p* < 0.01. * = Significance at the level *p* < 0.05.

**Table 6:** Association between anxiety, self-esteem and happiness index and behavior associated with cognition in the school context.

**Variables**	Model 0		Model 1	
	Unadjusted Model		Adjusted Model	
	βᵢ	[95% CI]	βᵢ	[95% CI]
Anxiety				
Memory use in class				
*Very low*	2.80	[1.66; 395] **	2.45	[1.31; 3.60] **
*Moderate*	1.83	[0.63; 3.03] **	1.61	[0.41; 2.81] **
*Severe*	Ref.		Ref.	
Speed to solve math problems				
*Very low*	0.85	[−0.55; 2.25]	0.13	[−1.21; 1.47]
*Moderate*	−0.36	[−1.83; 1.11]	−0.74	[−2.14; 0.66]
*Severe*	Ref.		Ref.	
Attention span				
*Very low*	1.47	[0.13; 2.81] *	1.08	[−0.26; 2.42]
*Moderate*	0.74	[−0.66; 2.15]	0.50	[−0.90; 1.91]
*Severe*	Ref.		Ref.	
Solution of complex tasks				
*Very low*	1.58	[0.27; 2.89] *	1.09	[−0.20; 2.39]
*Moderate*	0.68	[−0.69; 2.06]	0.43	[−0.93; 1.78]
*Severe*	Ref.		Ref.	
Nervousness during testing				
*Very low*	0.85	[−0.74; 2.43]	0.51	[−1.06; 2.09]
*Moderate*	−0.17	[−1.84; 1.49]	−0.35	[−1.99; 1.30]
*Severe*	Ref.		Ref.	
Self-esteem				
Memory use in class				
*Low perception*	−1.44	[−1.82; −1.06] **	−1.32	[−1.70; −0.94] **
*Moderate perception*	−0.62	[−0.87; −0.28] **	−0.57	[−0.92; −0.23] **
*High perception*	Ref.		Ref.	
Speed to solve math problems				
*Low perception*	−1.66	[−2.12; −1.20] **	−1.40	[−1.84; −0.96] **
*Moderate perception*	−0.86	[−1.28; −0.44] **	−0.72	[−1.12; −0.33] **
*High perception*	Ref.		Ref.	
Attention span				
*Low perception*	−1.77	[−2.21; −1.34] **	−1.66	[−2.09; −1.22] **
*Moderate perception*	−0.73	[−1.13; −0.34] **	−0.68	[−1.08; −0.29] **
*High perception*	Ref.		Ref.	
Solution of complex tasks				
*Low perception*	−1.48	[−1.91; −1.05] **	−1.31	[−1.74; −0.89] **
*Moderate perception*	−0.79	[−1.19; −0.40] **	−0.71	[−1.09; −0.32] **
*High perception*	Ref.		Ref.	
Nervousness during testing				
*Low perception*	−0.83	[−1.37; −0.30] **	−0.71	[−1.24;−0.18] **
*Moderate perception*	−0.39	[−0.87; 0.10]	−0.35	[−0.83; 0.13]
*High perception*	Ref.		Ref.	
Happiness				
Memory use in class				
*Low perception*	−1.45	[−1.87; −1.02] **	−1.32	[−1.74;−0.89] **
*Moderate perception*	−0.24	[−0.58; 0.10]	−0.26	[−0.60; 0.08]
*High perception*	Ref.		Ref.	
Speed to solve math problems				
*Low perception*	−1.53	[−2.04; −1.01] **	−1.27	[−1.76; −0.77] **
*Moderate perception*	−0.55	[−0.97; −0.14] **	−0.63	[−1.03; −0.24] **
*High perception*	Ref.		Ref.	
Attention span				
*Low perception*	−1.55	[−2.04; −1.06] **	−1.43	[−1.92; −0.93] **
*Moderate perception*	−0.59	[−0.98; −0.20] **	−0.63	[−1.03; −0.24] **
*High perception*	Ref.		Ref.	
Solution of complex tasks				
*Low perception*	−1.64	[−2.12; −1.16] **	−1.46	[−1.94; −0.99] **
*Moderate perception*	−0.63	[−1.02; −0.25] **	−0.66	[−1.04; −0.28] **
*High perception*	Ref.		Ref.	
Nervousness during testing				
*Low perception*	−0.87	[−1.47; −0.28] **	−0.70	[−1.29; −0.11] *
*Moderate perception*	−0.15	[−0.62; 0.33]	−0.19	[−0.66; 0.28]
*High perception*	Ref.		Ref.	

Data presented as β-coefficient and its 95% CI by anxiety, self-esteem and happiness category estimated by linear regression analysis. Severe anxiety, high perception of self-esteem and happiness were considered as reference values (Ref). Statistical analyses were incrementally adjusted: Model 0—unadjusted; Model 1—adjusted socio-educational variables (sex, school grade and special needs integration program). ** = Significance at the level *p* < 0.01. * = Significance at the level *p* < 0.05.

**Table 7:** Association between anxiety, self-esteem and happiness index and behavior associated with grade point average (GPA).

**Variables**	Model 0		Model 1	
	Unadjusted Model		Adjusted Model	
	βᵢ	[95% CI]	βᵢ	[95% CI]
Anxiety				
Math (1.0–7.0)				
*Very low*	0.59	[0.04; 1.15] *	0.32	[−0.22; 0.87]
*Moderate*	0.37	[−0.21; 0.95]	0.17	[−0.40; 074]
*Severe*	Ref.		Ref.	
Language (1.0–7.0)				
*Very low*	0.18	[−0.27; 0.63]	0.04	[−0.40; 0.48]
*Moderate*	0.11	[−0.36; 0.59]	0.02	[−0.44; 0.48]
*Severe*	Ref.		Ref.	
Physical education (1.0–7.0)				
*Very low*	0.41	[0.11; 0.70] **	0.23	[−0.05; 0.51]
*Moderate*	0.23	[−0.08; 0.54]	0.10	[−0.20; 0.39]
*Severe*	Ref.		Ref.	
Cumulative GPA (1.0–7.0)				
*Very low*	0.24	[−0.09; 0.56]	0.04	[−0.28; 0.35]
*Moderate*	0.08	[−0.26; 0.43]	−0.08	[−0.41; 0.25]
*Severe*	Ref.		Ref.	
Self-esteem				
Math (1.0–7.0)				
*Low perception*	−0.47	[−0.66; −0.29] **	−0.38	[−0.56; −0.20] **
*Moderate perception*	−0.22	[−0.39; −0.05] **	−0.17	[−0.33; −0.01] *
*High perception*	Ref.		Ref.	
Language (1.0–7.0)				
*Low perception*	−0.38	[−0.52; −0.23] **	−0.34	[−0.49; −0.20] **
*Moderate perception*	−0.28	[−0.42; −0.15] **	−0.26	[−0.40; −0.13] **
*High perception*	Ref.		Ref.	
Physical education (1.0–7.0)				
*Low perception*	−0.18	[−0.28; −0.08] **	−0.12	[−0.22; −0.03] *
*Moderate perception*	−0.07	[−0.16; 0.02]	−0.04	[−0.12; 0.05]
*High perception*	Ref.		Ref.	
Cumulative GPA (1.0–7.0)				
*Low perception*	−0.31	[−0.42; −0.21] **	−0.25	[−0.36; −0.15] **
*Moderate perception*	−0.23	[−0.33; −0.13] **	−0.20	[−0.29; −0.11] **
*High perception*	Ref.		Ref.	
Happiness				
Math (1.0–7.0)				
*Low perception*	−0.18	[−0.39; 0.03]	−0.10	[−0.30; 0.11]
*Moderate perception*	−0.06	[−0.23; 0.11]	−0.09	[−0.25; 0.07]
*High perception*	Ref.		Ref.	
Language (1.0–7.0)				
*Low perception*	−0.14	[−0.30; 0.03]	−0.09	[−0.25; 0.08]
*Moderate perception*	−0.14	[−0.27; 0.00]	−0.12	[−0.25; 0.01]
*High perception*	Ref.		Ref.	
Physical education (1.0–7.0)				
*Low perception*	−0.18	[−0.29; −0.07] **	−0.11	[−0.22; −0.01] *
*Moderate perception*	−0.02	[−0.11; 0.07]	−0.03	[−0.12; 0.05]
*High perception*	Ref.		Ref.	
Cumulative GPA (1.0–7.0)				
*Low perception*	−0.17	[−0.29; −0.04] **	−0.10	[−0.22; 0.01]
*Moderate perception*	−0.11	[−0.20; −0.01] *	−0.11	[−0.21; −0.02] *
*High perception*	Ref.		Ref.	

Data presented as β-coefficient and its 95% CI by anxiety, self-esteem and happiness category estimated by linear regression analysis. Severe anxiety, high perception of self-esteem and happiness were considered as a reference value (Ref). Statistical analyses were incrementally adjusted: Model 0—unadjusted; Model 1—adjusted socio-educational variables (sex, school grade and special needs integration program). ** = Significance at the level *p* < 0.01. * = Significance at the level *p* < 0.05.

## Data Availability

Data will be made available upon request.
